# Non-canonical NF-κB signaling in rheumatoid arthritis: Dr Jekyll and Mr Hyde?

**DOI:** 10.1186/s13075-015-0527-3

**Published:** 2015-01-28

**Authors:** Ae R Noort, Paul P Tak, Sander W Tas

**Affiliations:** Department of Clinical Immunology & Rheumatology, Academic Medical Center/University of Amsterdam, 1105AZ Amsterdam, The Netherlands; Department of Experimental Immunology, Academic Medical Center/University of Amsterdam, 1105 AZ Amsterdam, The Netherlands; Department of Medicine, University of Cambridge, Cambridge, CB2 1TN UK; Current address: GlaxoSmithKline, Stevenage, SG1 2NY UK

## Abstract

The nuclear factor-κB (NF-κB) family of transcription factors is essential for the expression of pro-inflammatory cytokines, but can also induce regulatory pathways. NF-κB can be activated via two distinct pathways: the classical or canonical pathway, and the alternative or non-canonical pathway. It is well established that the canonical NF-κB pathway is essential both in acute inflammatory responses and in chronic inflammatory diseases, including rheumatoid arthritis (RA). Although less extensively studied, the non-canonical NF-κB pathway is not only central in lymphoid organ development and adaptive immune responses, but is also thought to play an important role in the pathogenesis of RA. Importantly, this pathway appears to have cell type-specific functions and, since many different cell types are involved in the pathogenesis of RA, it is difficult to predict the net overall contribution of the non-canonical NF-κB pathway to synovial inflammation. In this review, we describe the current understanding of non-canonical NF-κB signaling in various important cell types in the context of RA and consider the relevance to the pathogenesis of the disease. In addition, we discuss current drugs targeting this pathway, as well as future therapeutic prospects.

## Introduction

### Rheumatoid arthritis

Rheumatoid arthritis (RA) is a disabling chronic inflammatory autoimmune disease affecting the synovial joints. In the early phase of the disease, the synovial tissue is infiltrated by immune cells and increases in thickness, which causes pain, stiffness and swelling of the joint. The synovial cell infiltrate contains various lymphocytes, plasma cells, macrophages, and other cells. These cells contribute to the inflammatory process via the production of matrix metalloproteinases (MMPs), cytokines and chemokines, followed by the influx and activation of more immune cells into the synovial tissue. From the earliest stage of the disease, neoangiogenesis can be observed, which contributes to chronicity. Eventually, the loss of articular cartilage, along with damage to the joint capsule and peri-articular structures, causes deformities (reviewed in [1]).

In RA synovial tissue many signal transduction pathways are activated [2]. One of the most important signaling pathways involved in the pathogenesis of RA is the nuclear factor-κB (NF-κB) pathway (reviewed in [3]).

### Nuclear factor-κB

NF-κB is expressed ubiquitously in the cytoplasm of almost all cell types. Many diseases, including cancer, and inflammatory and autoimmune diseases, are associated with dysregulation of NF-κB (reviewed in [4]). NF-κB can be activated via two distinct pathways, the classical or canonical NF-κB pathway, and the alternative or non-canonical NF-κB pathway.

#### The canonical NF-κB pathway

**Figure 1:**
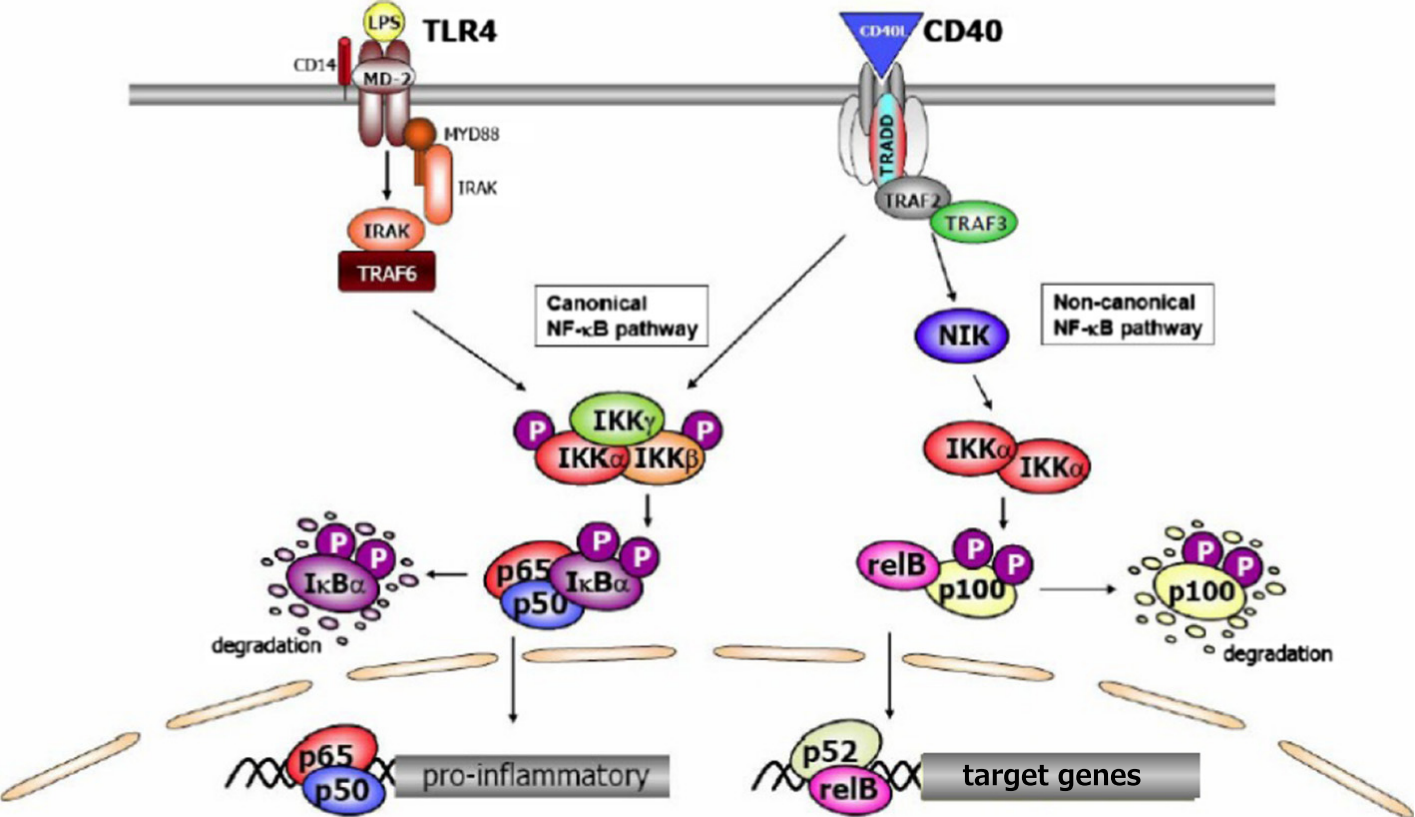
**Overview of nuclear factor-κ** **B activation pathways.** Schematic representation of the canonical and non-canonical nuclear factor (NF)-κB pathways. The canonical NF-κB pathway can be activated by a variety of different stimuli, like tumor necrosis factor-α and lipopolysaccharide (LPS). Activation of the canonical pathway via Toll-like receptor or cytokine receptor signaling depends on the inhibitor of κB kinase (IKK) complex, which is composed of the kinases IKKα and IKKβ, and the regulatory subunit IKKγ (NEMO). Activated IKK phosphorylates the inhibitory subunit IκBα to induce its degradation, allowing NF-κB dimers (p50-p65) to translocate to the nucleus and bind to DNA to induce NF-κB target gene transcription. The non-canonical pathway (right) is activated by specific stimuli like B cell activating factor, lymphotoxin β, LIGHT and CD40L. NF-κB inducing kinase (NIK) is stabilized and activates and recruits IKKα into the p100 complex to phosphorylate p100, leading to p100 ubiquitination. Processing of p100 generates the p52/RelB NF-κB complex, which is able to translocate to the nucleus and induce gene expression.

The most extensively studied NF-κB activation pathway is the canonical pathway (Figure 1), which can be activated by stimulation of a variety of cell membrane receptors, including tumor necrosis factor (TNF) receptor, interleukin (IL)-1 receptor, and Toll-like receptors, in response to pro-inflammatory stimuli like lipopolysaccharide, IL-1 and TNF, as well as via triggering of the T-cell receptor or B-cell receptor. In this pathway, inhibitor of κB kinase (IKK)β is required for NF-κB activation, whereas IKKα is redundant [4]. The canonical NF-κB pathway is essential both in acute inflammatory responses and in chronic inflammatory diseases such as RA and inflammatory bowel disease. Moreover, this pathway is important in cell proliferation and survival, demonstrated by constitutively active NF-κB signaling in many tumor tissues [5]. In RA IKKβ is a key regulator of synovial inflammation [6] and the importance of the canonical NF-κB pathway in arthritis is underlined by the beneficial effects of specific IKKβ inhibition in preclinical models of arthritis [6,7] and the common and successful use of anti-TNF therapy in RA, one of the main target genes of the canonical NF-κB pathway. This is outside the scope of the current review, however, but is discussed in more detail in previous reviews [2,3]. Here we focus on the alternative or non-canonical NF-κB pathway.

#### The non-canonical NF-κB pathway

In the past decade, a second, alternative NF-κB activation pathway was identified, the so-called non-canonical NF-κB pathway (Figure 1). This pathway can be triggered by the activation of members of the TNF-receptor superfamily including the lymphotoxin β (LTβ) receptor (LTβR), CD40, B cell activating factor (BAFF) belonging to the TNF family receptor, and receptor activator of NF-κB (RANK). Of note, these receptors not only trigger the non-canonical NF-κB pathway, but simultaneously also the canonical pathway. The non-canonical NF-κB pathway is strictly dependent on IKKα homodimers and unlike the canonical pathway does not involve IKKβ or IKKγ [8]. In the steady state, NF-κB inducing kinase (NIK), the most important kinase of the non-canonical pathway, is continuously degraded. TRAF3 mediates recruitment of NIK to TRAF2, which leads to ubiquitination of NIK. Consequently, endogenous levels of NIK are very low and the NF-κB complex is retained in the cytoplasm and kept inactive. Upon signal-induced activation of the non-canonical NF-κB pathway, TRAF2 induces proteolysis of TRAF3. Degradation of TRAF3 prevents targeting of newly synthesized NIK, resulting in accumulation of NIK. Subsequently, NIK induces processing of p100 by IKKα homodimers, resulting in partial degradation into p52. Next, mainly p52-RelB heterodimers translocate to the nucleus, leading to target gene transcription. Whereas canonical NF-κB activation is rapid and independent of protein synthesis, non-canonical NF-κB activation requires NIK synthesis and accumulation. Consequently, the kinetics of this pathway are considerable slower (reviewed in [4,8]).

Of note, ligands of the TNF-receptor superfamily simultaneously also activate the canonical pathway and regulated cross-talk between both pathways exists at different levels. IKKα has, for instance, been described to also have nuclear functions and serves as a regulator of canonical NF-κB-dependent gene expression through control of promoter-associated histone phosphorylation after cytokine exposure [9,10]. In macrophages, however, IKKα also terminates canonical NF-κB-dependent transcription of target genes by accelerating both the turnover of the NF-κB subunits RelA and c-Rel and their removal from pro-inflammatory gene promoters [11]. Reciprocally, canonical NF-κB activity suppresses basal non-canonical NF-κB signaling in immune cells [12]. Interestingly, it has been shown that, under certain circumstances, other stimuli, including TNF, can to some extent also activate non-canonical NF-κB signaling in specific cell types [13], and IKKα is critical for interferon-α production induced by Toll-like receptors 7 and 9 [14]. The functional role of the non-canonical NF-κB pathway *in vivo* has been established by the identification of phenotypic abnormalities shared by knockout mice or mutants of specific signaling molecules involved in this pathway. Overall, this pathway is crucially involved in lymphoid organ development and adaptive immune responses [8]. The role of non-canonical NF-κB signaling in (synovial) inflammation is addressed below.

## Non-canonical NF-κB signaling in rheumatoid arthritis

In the inflamed RA synovial tissue, all stimuli that are able to induce non-canonical NF-κB signaling are abundantly present. LTβ plays an essential role in lymphoid organ development and it has been demonstrated that this is dependent on LTβR-induced non-canonical NF-κB activation (reviewed in [15]). In RA synovial tissue, tertiary lymphoid structures (TLSs) can be formed that often resemble germinal centers with organized B- and T-cell clusters. Of note, LTβ is also important in the formation of TLSs in RA synovitis [16]. Signaling through the LTβR can also be induced by LIGHT and high levels of LIGHT have been observed in serum, synovial tissue, as well as in synovial fluid of RA patients [17]. In addition, LIGHT is upregulated on B cells and monocytes in RA peripheral blood and synovial fluid [18].

Activation of the non-canonical NF-κB pathway can also be induced via BAFF/BAFF receptor (BAFF-R) or CD40L/CD40 triggering. CD40L is highly expressed in RA synovial tissue [19], and elevated in serum and peripheral blood of RA patients [20]. Furthermore, CD40L and CD40 are highly expressed by T lymphocytes and macrophages in the synovial fluid of RA patients [21]. In patients with RA, elevated serum levels of BAFF have been observed [22], and BAFF/BAFF-R are widely expressed in RA synovium [23].

Interaction of RANK and RANK ligand (RANKL) has been extensively studied in RA because of the important role in osteoclast biology. RANKL is highly expressed in synovial tissue of RA patients with active disease [24] and increased levels of soluble RANKL are found both in serum and synovial fluid from RA patients [25].

Together, these studies suggest that activators of the non-canonical NF-κB pathway play an important role in RA synovial inflammation, although direct involvement of the non-canonical NF-κB pathway is hitherto lacking. Nik^−/−^ mice and aly/aly mice that are homozygous for a spontaneous recessive point mutation in the gene encoding NIK both lack lymph nodes and have a disturbed microarchitecture of the spleen. Furthermore, these mice have reduced humoral and cell-mediated immune functions, and are more susceptible to infections, although they do have mature T and B cells [26,27]. Relb^−/−^ mice develop a complex phenotype, including an autoimmune-like inflammatory syndrome, myeloid hyperplasia, multifocal defects in immune responses, and impaired development of lymphoid organs [28-30]. These studies demonstrate that the non-canonical NF-κB pathway controls the expression of genes involved in B-cell function and lymphoid organogenesis, as well as cell proliferation and survival. NIK has also been shown to be critical for inflammation-induced osteoclastogenesis, as NIK-deficient mice were largely resistant to arthritis with less osteoclastogenesis and less bone erosion [31]. Importantly, the non-canonical NF-κB pathway appears to play different roles in different cell types. Since many cell types are involved in the pathogenesis of RA, it is very difficult to predict the net overall contribution of non-canonical NF-κB signaling to synovial inflammation. Therefore, the role of the non-canonical NF-κB pathway in the most important cell types involved in RA synovial inflammation is discussed below.

### Macrophages

Inflamed RA synovial tissue contains many macrophages that contribute to inflammation, which is substantiated by our observation that the number of CD68^+^ synovial sublining macrophages correlates with clinical improvement independent of therapeutic strategy [32]. However, macrophages are phenotypically heterogeneous, and are not only the main producers of proinflammatory cytokines like TNF, IL-1, IL-15, IL-18, IL-23, and IL-27 that play an important role in the persistence of inflammation, but also generate inhibitory mediators such as IL-10, transforming growth factor-β, and soluble TNF receptor (reviewed in [33]). We and others have demonstrated that the canonical NF-κB pathway plays an important role in macrophage production of TNF-α and destructive MMPs [7]. The non-canonical NF-κB pathway is activated during human monocyte-macrophage differentiation and the increase in IKKα may act as a brake preventing hyperactivation of the new macrophages [34]. IKKα accelerates turnover of the classical NF-κB subunits RelA and c-Rel in macrophages, and their removal from pro-inflammatory gene promotors, thereby negatively regulating its own activation. Consequently, inactivation of IKKα in mice resulted in enhanced inflammation [11]. These data strongly suggest that the non-canonical NF-κB pathway plays a regulatory role in macrophages. In contrast to these findings, non-canonical NF-κB signaling in macrophages is critical for production of the chemokine CXCL12, which is required for cells to migrate toward HMGB1, a proinflammatory cytokine and chemoattractant for immune effector cells [35].

The abundant presence of non-canonical NF-κB ligands in RA synovial tissue is likely to induce signaling in macrophages as well. LIGHT is expressed in CD68^+^ macrophages and *in vitro* stimulation of these cells with LIGHT induced expression of MMP-9 and pro-inflammatory cytokines TNF-α, IL-6, and IL-8 [36]. Synovial fluid macrophages express increased levels of CD40 and produce both IL-12p40, TNF-α and IL-10 after CD40 ligation [37]. However, the contribution of the non-canonical NF-κB pathway to these LIGHT- and CD40L-induced processes remains to be tested. In contrast to other leukocytes, macrophages are more long-lived cells and also persist at inflammatory lesions during the resolution of inflammation [38]. Therefore, the non-canonical NF-κB pathway may play a dual role in macrophages and can act as both a pro-inflammatory and an anti-inflammatory pathway.

### Dendritic cells

In RA synovial tissue, immature and mature dendritic cell (DC) subsets can be observed in close association with T cells and B-cell follicles [39]. These DCs may contribute to ongoing inflammation through presentation of autoantigens or production of pro-inflammatory cytokines. NF-κB is essential for normal DC differentiation, activation and survival [40]. CD40 ligation on DCs induces early production of inflammatory cytokines via the canonical NF-κB pathway, as well as late expression of the anti-inflammatory enzyme indoleamine 2,3-dioxygenase via non-canonical NF-κB signaling, which is able to suppress T-cell activation and to promote T cells with regulatory functions [41]. Interestingly, both synovial fluid and synovial tissues of RA patients contain DCs that express functional indoleamine 2,3-dioxygenase [42], pointing towards a possible mechanism of tolerance in RA synovial inflammation. These results are consistent with *in vivo* experiments performed in mice lacking functional NIK that have decreased numbers of DCs which also have lower expression of costimulatory molecules and exhibit reduced antigen presentation. Consequently, these DCs have a decreased ability to induce expansion of CD25^+^CD4^+^ regulatory T cells [43]. At the same time, it was demonstrated that DCs require non-canonical NF-κB signaling to cross-prime CD8^+^ T cells [44]. Furthermore, NIK expression in mouse DCs is also important for supplying co-stimulatory signals to CD4^+^ T cells [45]. In human DCs, however, NIK was not essential for effective antigen presentation [46].

Taken together, the non-canonical NF-κB pathway regulates both pro-inflammatory and anti-inflammatory processes in DCs. The net result is likely to rely heavily on additional stimuli and the microenvironment in which the cells are present.

### B cells and plasma cells

B cells are present in the inflamed RA synovial tissue and the efficacy of anti-CD20 treatment has confirmed the pivotal role of B cells in the pathogenesis of RA in patients [47]. The canonical NF-κB pathway was originally described in B cells and is crucial for B-cell development, maintenance, and function (reviewed in [48]). However, the non-canonical pathway also plays an important role in B-cell biology. IKKα in B cells is crucial for germinal center formation, and long-lived immunoglobin titers, but not for primary antibody production [49]. Importantly, NIK also regulates BAFF-mediated expression of inducible costimulator ligand, a molecule required for follicular helper T cell generation [50]. In addition, NIK provides survival signals in B cells, which is illustrated by the fact that B cell-specific ablation of TRAF2 and TRAF3 (negative regulators of NIK) markedly enhanced the survival and proliferation of B cells [51]. Furthermore, non-canonical NF-κB signaling also regulates plasma cell generation, proliferation and survival [52]. In conclusion, the non-canonical pathway plays an important role in B cells and plasma cells by promoting survival, differentiation and antibody production, which is likely to contribute to the persistence of inflammation in RA.

### T cells

Many T cell subsets can be found in RA synovial tissue, including Th1, Th17 and regulatory T cells (reviewed in [53]). The canonical NF-κB pathway is well known for its role in the activation and differentiation of T cells [54]. In contrast, the role of the non-canonical NF-κB pathway in T-cell function is less well studied. It has been shown that NIK and RelB are essential for T-cell activation via the T-cell receptor/CD3 pathway [55]. NIK and IKKα have a T-cell-intrinsic function in the regulation of Th17 cells, a subset of CD4^+^ effector T cells that produce the IL-17 family of cytokines, which are intimately involved in autoimmunity, including in RA [56,57]. The non-canonical NF-κB pathway is also required for the generation of CD4^+^ T-follicular helper cells mediating B-cell activation and antibody responses [50]. In contrast, NIK is essential for the generation of regulatory T cells that dampen inflammatory responses as well [58].

In summary, the non-canonical NF-κB pathway has dual roles in T cells; it is not only important in T-cell activation and the induction of Th17 cells, but is also required for the generation of regulatory T cells.

### Osteoclasts

In RA synovial tissue, osteoclasts that resorb bone are found at sites adjacent to bone, causing local bone destruction [59]. Interestingly, aly/aly mice that lack functional NIK have increased bone mineral density and bone volume. In addition, these mice and Nik^−/−^ mice have a significant defect in RANKL-induced osteoclastogenesis *in vitro* and *in vivo* [60,61]. Also, Nik^−/−^ mice exhibit significantly less periarticular osteoclastogenesis and less bone erosion in the serum transfer arthritis model [31]. Interestingly, overexpression of constitutively active IKKα or p52 restored osteoclastogenesis in aly/aly cells [60] and re-introduction of RelB, but not p65, in relb^−/−^ cells rescued osteoclast formation [62]. Activated osteoclasts are responsible for the bone loss associated with RA. Constitutive activation of NIK drives enhanced osteoclastogenesis and bone resorption, both in basal conditions and in response to inflammatory stimuli [63]. In humans, RANKL expressed by synovial fibroblasts [64] or T cells [65] effectively induces osteoclastogenesis. Also, IKKα is required for RANKL-induced osteoclast formation *in vitro* [66]. In conclusion, non-canonical NF-κB signaling in osteoclasts unambiguously contributes to bone destruction in RA, suggesting that this pathway may be an interesting target in these cells.

### Synovial fibroblasts

RA synovial fibroblasts (RASFs) create an abnormal stromal microenvironment that is thought to be crucial for the persistence of inflammation, not only because of its architecture, but also by secreting pro-inflammatory cytokines or by producing growth factors that stimulate neovascularization. In addition, it has been demonstrated that RASFs display certain unique features, such as an invasive, tumor-like behavior that is normally not observed in fibroblasts (reviewed in [67]). Non-canonical NF-κB signaling contributes to inflammation in RASFs since NIK is essential for LTβR activation of NF-κB in these cells [68]. Also, stromal cells from mice lacking functional NIK showed decreased levels of adhesion molecules and increased CXCL13 expression. These findings suggest that NIK activity in stromal cells may play an important role in regulating the migration of immune cells [69]. Stimulation of RASFs with LIGHT results in upregulation of adhesion molecules and MMPs [18]. Also, CD40 ligation of RASFs induces RANKL expression, resulting in enhanced osteoclast formation [64]. Stimulation of RASFs with CD40L also induces the non-canonical pathway target gene *CXCL12*, which may promote angiogenesis and the migration of T cells, B cells, and monocytes/macrophages into the inflamed synovium [70].

In summary, non-canonical NF-κB signaling in RASFs contributes to the pathological behavior of these cells, leading to persistence of inflammation.

### Endothelial cells

Endothelial cells (ECs) play a crucial role in the pathogenesis of RA. First, they express adhesion molecules and produce chemokines, thereby acting as the site of entry for immune cells into the synovial tissue. In addition, ECs proliferate and give rise to neovascularization that contributes to the persistence of inflammation (reviewed in [71]). In synovial tissue containing TLSs, highly differentiated ECs, such as high endothelial venules, can also be observed [16]. Consequently, the signaling pathways that control activation of ECs have been extensively studied and the canonical NF-κB pathway was demonstrated to play an important role in the expression of many of the pro-inflammatory genes [72]. Madge and colleagues [73] reported that LIGHT and LTα1β2, but not TNF, induce CXCL12 expression in ECs, which required non-canonical NF-κB signaling. CXCL12 expression in ECs has been demonstrated in RA synovial tissue as well [74]. In addition, CXCL12 colocalized with α_v_β_3_, a marker for neoangiogenesis, in RA ECs, which would suggest that CXCL12 may be involved in RA synovial tissue angiogenesis [74]. We have observed that CXCL12 is expressed by NIK^+^ blood vessels in RA synovial tissue. Subsequently, we established that non-canonical NF-κB signaling in ECs stimulates pathological angiogenesis [75]. Recently, it was demonstrated that EC-specific LTβR signaling is crucial for lymph node and high endothelial venule formation in mice, designating ECs as an important player in organizing lymphoid tissue [76]. Unpublished results from our group further support these data, as we observed high numbers of NIK^+^ ECs in TLSs in RA synovial tissue and correlated this with the presence of perivascular (pre-) follicular dendritic cells. This suggests that these cells may be important in the formation of TLSs in chronic inflammation (Noort *et al*., unpublished observations).

In summary, non-canonical NF-κB signaling in ECs is likely to contribute to angiogenesis and the influx of immune cells into the inflamed RA synovial tissue, thereby perpetuating the inflammatory response. Therefore, blocking this pathway in ECs is probably beneficial in RA.

## Targeting the non-canonical NF-κB pathway in rheumatoid arthritis

Despite the diverse roles of the non-canonical NF-κB pathway in different cell types, overall this pathway is likely to contribute to the persistence of inflammation in RA. As alluded to earlier, non-canonical NF-κB signaling can be induced via triggering of various TNF-receptor superfamily members, such as LTβR, CD40, BAFF-R, and RANK, by their respective ligands. The important role of these ligand-receptor pairs in (synovial) inflammation is illustrated by the numerous attempts of pharmaceutical companies to target these pathways. Below, we will discuss compounds that have been tested in RA patients or preclinical models of RA (Table 1).

**Table 1:** **Biologics targeting ligand-receptor pairs involved in non-canonical nuclear factor-**κ**B signaling in rheumatoid arthritis**

**Ligand**	**Receptor**	**Biologic**	**Type of agent**	**Activity of biologic**	**Stage of development**
BAFF	BAFF-R	Belimumab (Benlysta; Human Genome Sciences/GlaxoSmithKline)	Human BAFF-specific antibody	Antagonist	Phase II
		Tabalumab/LY2127399	Human BAFF-specific antibody	Antagonist	Phase II
CD40L	CD40	BI 655064	Humanized CD40-specific antibody	Antagonist	Phase I in progress
-	LTβR	Baminercept	Human LTβR-IgG_1_ fusion protein	Antagonist	Phase II
LTα	LTβR	Patecluzimab	Humanized LTα-specific antibody	Depleting and antagonist	Phase II in progress
RANKL	RANK	Denosumab (Prolia/Xgeva; Amgen)	Human RANKL-specific antibody	Antagonist	Phase II

BAFF, B cell activating factor; BAFF-R, BAFF receptor; CD40L, CD40 ligand; Ig, immunoglobulin; LTα, lymphotoxin-α; LTβR, LTβ receptor; RANK, receptor activator of nuclear factor-κB; RANKL, RANK ligand.

### Anti-BAFF/BAFF-R treatment

Belimumab, a fully human IgG1 monoclonal antibody to BAFF, was tested in RA in a phase II placebo-controlled dose-ranging trial. Due to the modest clinical response in this trial, it was not further tested for RA [77]. However, belimumab has been proven to have a beneficial clinical effect in systemic lupus erythematosus (SLE) patients and is licensed for use in SLE in the United States, Canada, and Europe. A different anti-BAFF monoclonal antibody, tabalumab, has also been tested in patients with active RA. This drug showed modest efficacy [78], but nevertheless clinical development in RA was discontinued.

### Anti-CD40/CD40L treatment

Clinical trials targeting the CD40/CD40L interaction in RA are currently ongoing, but so far have not been published. However, animal models have demonstrated encouraging results. In the collagen-induced arthritis (CIA) model, treatment with agonistic CD40 antibodies at the time of CIA induction exacerbates disease [79]. In contrast, treatment with anti-CD40L antibodies prior to CIA induction ameliorated development of disease [80], resulting in less synovial inflammation, and less bone erosion [81]. In humans, two clinical studies investigating the effects of anti-CD40L treatment have been conducted in patients with SLE. Both ruplizumab and toralizumab showed promising clinical and laboratory responses in some SLE patients. However, one of these studies was discontinued prematurely because of thromboembolic events [82]. Since then, new reagents inhibiting CD40L-mediated events that are less likely to increase the risk of thromboembolic complications have been developed and clinical trials in RA patients are in progress.

### Anti-LTβ/LTβR treatment

In the collagen-induced arthritis model, treatment with a LTβR-Ig fusion protein resulted in reductions in the severity of disease and joint tissue damage [83].

The efficacy and safety of baminercept, a LTβR-IgG_1_ fusion protein, was evaluated in patients with RA in a placebo-controlled, phase IIb trial [84]. However, baminercept did not exhibit measurable clinical effects in RA patients. Pateclizumab, a humanized mouse antibody that specifically binds LTα in both the soluble LTα3 homotrimeric form and the surface-expressed LTα1β2 heterotrimer, thereby interfering with binding of LT trimers to their cognate receptors, was tested for safety and efficacy in a phase I study. Preliminary beneficial effects were observed in RA patients [85] and a phase II trial to further test pateclizumab is now in progress.

### Anti-RANK/RANKL treatment

In mice, treatment with a neutralizing anti-RANKL monoclonal antibody in CIA resulted in amelioration of bone loss [86]. Phase II clinical trials suggest that denosumab-mediated inhibition of RANKL in RA patients prevents bone loss at the site of inflammation, but has no apparent effect on inflammation [87]. Nevertheless, denosumab could be used as an adjunctive therapy next to established (biologic) disease-modifying antirheumatic drugs to prevent structural joint damage in RA.

One has to bear in mind that virtually all stimuli that activate TNF receptor superfamily members and induce non-canonical pathway signaling simultaneously also activate the canonical pathway. Hence, determining whether the observed therapeutic effects of blocking these receptor-ligand pairs are mediated via inhibition of the non-canonical pathway or can also be attributed to effects on canonical NF-κB signaling is difficult and requires further investigation.

### Future perspectives: direct targeting of intracellular signaling molecules

Rather than indirect inhibition of non-canonical NF-κB signaling using the compounds described above, direct inhibition of NIK or IKKα may be more effective. In RA synovial inflammation, NIK can be targeted using intra-articular gene therapy (Noort *et al*., unpublished, ongoing studies in preclinical models of arthritis) or using small molecule inhibitors. Since NIK levels in normal cells are usually low, it is anticipated that therapeutics designed to limit the amount of NIK will not cause serious side effects. Consequently, NIK inhibition using specific small molecule inhibitors could perhaps be an effective treatment option not only for RA but also for other chronic inflammatory diseases. Recently, a report was published in which the discovery, structure-based design, synthesis, and optimization of several selective NIK inhibitors are described [88]. So far, only one specific IKKα inhibitor, BAY32-5915, has been reported [89]. We expect that preclinical studies will soon answer the question of whether selective targeting of NIK or IKKα and consecutive non-canonical NF-κB signaling may be beneficial in (rheumatoid) arthritis as well. Alternatively, in some cell types it may be beneficial to actually stimulate non-canonical NF-κB activity to obtain a therapeutic effect. Smac mimetics or inhibitor of apoptosis protein antagonists that promote degradation of cIAP1 and cIAP2 are not only able to induce apoptosis in cancer cells, but also promote stabilization of NIK and consequent activation of the non-canonical NF-κB pathway in resting cells [90,91]. Currently, one of these smac mimetic compounds, called Birinapant, is undergoing clinical development for the treatment of solid tumors and hematological malignancies [92,93].

## Conclusion

**Figure 2:**
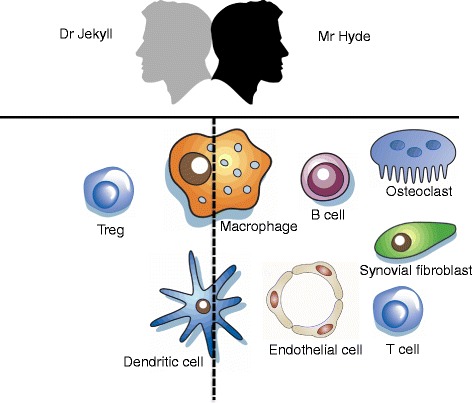
**Non-canonical nuclear factor-** **κ** **B signaling in different cell types in rheumatoid arthritis: Dr Jekyll or Mr Hyde?** Schematic overview of the role of the non-canonical nuclear factor (NF)-κB pathway in cell types involved in the pathogenesis of rheumatoid arthritis. On the left side (Dr Jekyll), cells in which non-canonical NF-κB signaling plays an anti-inflammatory role. In the middle (on the dashed line), cells in which this pathway plays a dual role, which is largely dependent on the microenvironment. On the right side (Mr Hyde), cells in which non-canonical NF-κB signaling is pro-inflammatory. Treg, regulatory T cell.

The studies reviewed above support the role of the non-canonical NF-κB pathway as a major participant in inflammatory responses in general and the pathogenesis of RA in particular. NIK and downstream non-canonical NF-κB signaling have diverse functions, depending on the cell types in which this pathway is activated. Roughly, in synovial fibroblasts, osteoclasts, endothelial cells, and B cells/plasma cells, non-canonical NF-κB signaling contributes to the inflammatory process by triggering the secretion of critical inflammatory mediators and matrix-degrading enzymes involved in tissue degradation, whereas in macrophages, dendritic cells and T cells this pathway has more pleiotropic functions and can also play a more regulatory, anti-inflammatory role (Figure 2). In these cells non-canonical NF-κB signaling shows clear resemblance with ‘*The Strange Case of Dr Jekyll and Mr Hyde*’ (Robert Louis Stevenson): there is a good and an evil side. Further elucidation of the precise function of this pathway in individual cell types will result in a better understanding of the overall contribution of the non-canonical NF-κB pathway to the complex cellular networks involved in RA synovial inflammation. This will allow the design of better therapeutic strategies for the management of this disease, including cell type-specific inhibitors or selective targeting of inhibitors to certain cell types. For this application, anti-DEC-205 antibodies may be used to target compounds specifically to DCs or to selectively target ECs, and (peptide) inhibitors can be coupled to a multimodular recombinant protein that specifically binds to cytokine-activated endothelium, which has been demonstrated to work very elegantly under inflammatory conditions *in vivo* [94].

Although the non-canonical NF-κB pathway has anti-inflammatory effects in some cell types, we believe that in chronic inflammation the positive effects of targeting the non-canonical NF-κB pathway will surpass the possible negative effects. We anticipate that the advent of several selective NIK inhibitors will aid in further establishing the non-canonical NF-κB pathway as a promising new therapeutic target, not only in RA, but also in other immune-mediated inflammatory diseases.

## Abbreviations

BAFF: B cell activating factor
BAFF-R: B cell activating factor receptor
CIA: Collagen-induced arthritis
DC: Dendritic cell
EC: Endothelial cell
IKK: Inhibitor of κB kinase
IL: Interleukin
LTβ: Lymphotoxin β
LTβR: Lymphotoxin β receptor
MMP: Matrix metalloproteinase
NF-κB: Nuclear factor-κB
NIK: NF-κB inducing kinase
RA: Rheumatoid arthritis
RANK: Receptor activator of NF-κB
RANKL: Receptor activator of NF-κB ligand
RASF: Rheumatoid arthritis synovial fibroblast
SLE: Systemic lupus erythematosus
TLS: Tertiary lymphoid structure
TNF: Tumor necrosis factor
